# Shotgun metagenomic sequencing revealed the prebiotic potential of a grain-based diet in mice

**DOI:** 10.1038/s41598-022-10762-3

**Published:** 2022-04-25

**Authors:** Aditi Jangid, Shinji Fukuda, Yutaka Suzuki, Todd D. Taylor, Hiroshi Ohno, Tulika Prakash

**Affiliations:** 1 BioX Centre and School of Basic Sciences, Indian Institute of Technology Mandi, Kamand, Mandi, Himachal Pradesh 175005 India; 2 Institute for Advanced Biosciences, Keio University, Tsuruoka, Yamagata, 997-0052 Japan; 3 Laboratory for Intestinal Ecosystem, RIKEN Center for Integrative Medical Sciences, Yokohama, Kanagawa 230-0045 Japan; 4 Gut Environmental Design Group, Kanagawa Institute of Industrial Science and Technology, Kawasaki, Kanagawa 210-0821 Japan; 5 Transborder Medical Research Center, University of Tsukuba, Tsukuba, Ibaraki 305-8575 Japan; 6 Department of Computational Biology and Medical Sciences, The University of Tokyo, 5-1-5, Kashiwanoha, Kashiwa, Chiba, 277-8562 Japan; 7 Laboratory for Microbiome Sciences, RIKEN Center for Integrative Medical Sciences, Tsurumi-ku, Yokohama, Kanagawa 230-0045 Japan

**Keywords:** Computational biology and bioinformatics, Immunology, Systems biology

## Abstract

In the present study, we elucidated the effect of grain-based (GB) diet containing both soluble and insoluble fibers and purified ingredients-based (PIB) diet containing only insoluble fiber, namely cellulose on mice gut microbiome using whole shotgun based metagenomic sequencing. Although the fiber content in both diet types is the same (5%) the presence of soluble fiber only in the GB diet differentiates it from the PIB diet. The taxonomic analysis of sequenced reads reveals a significantly higher enrichment of probiotic *Lactobacilli* in the GB group as compared to the PIB group. Further, the enhancement of energy expensive cellular processes namely, cell cycle control, cell division, chromosome partitioning, and transcription is observed in the GB group which could be due to the metabolization of the soluble fiber for faster energy production. In contrast, a higher abundance of cellulolytic bacterial community namely, the members of family *Lachnospiraceae* and *Ruminococcaceae* and the metabolism functions are found in the PIB group. The PIB group shows a significant increase in host-derived oligosaccharide metabolism functions indicating that they might first target the host-derived oligosaccharides and self-stored glycogen in addition to utilising the available cellulose. In addition to the beneficial microbial community variations, both the groups also exhibited an increased abundance of opportunistic pathobionts which could be due to an overall low amount of fiber in the diet. Furthermore, backtracing analysis identified probiotic members of *Lactobacillus*, viz., *L. crispatus ST1*, *L. fermentum CECT 5716*, *L. gasseri ATCC 33323*, *L. johnsonii NCC 533* and *L. reuteri 100-23* in the GB group, while *Bilophila wadsworthia 3_1_6*, *Desulfovibrio piger ATCC 29098*, *Clostridium symbiosum WAL-14163*, and *Ruminococcaceae bacterium D16* in the PIB group. These data suggest that *Lactobacilli*, a probiotic community of microorganisms, are the predominant functional contributors in the gut of GB diet-fed mice, whereas pathobionts too coexisted with commensals in the gut microbiome of the PIB group. Thus at 5% fiber, GB modifies the gut microbial ecology more effectively than PIB and the inclusion of soluble fiber in the GB diet may be one of the primary factors responsible for this impact.

## Introduction

A major difference has been observed in the dietary habits of the population living in industrialized countries as compared to the traditional agrarians. The westernized diet has a characteristic high content of protein and fats as opposed to the diets of the traditional societies which are rich in dietary fiber^1^. Numerous studies have demonstrated that the dietary fiber is a significant factor impacting the gut microbiome and intestinal health^2^. Fiber generally describes most carbohydrate polymers which escape digestion and absorption in the upper gastrointestinal (GI) tract^3^. These polymers reach the lower GI-tract, where members of the gut microbiome ferment them. These polymers either occur naturally in food or are synthesized by chemical, physical, or enzymatic methods^3^. Dietary fibers are classified as fermentable (soluble) and non-fermentable (insoluble) according to their fermentability^4^. The beneficial roles of fermentable fibers and their mechanisms have been well studied. Particularly, the short-chain fatty acids (SCFAs), such as acetate, butyrate, and propionate, which are the main products fermented from the soluble fiber by the commensal bacteria, are thought to play a pivotal role in the maintenance of intestinal immunity and health^5–7^.

For laboratory animals, the grain-based (GB) and purified ingredients-based (PIB) are the two most popular diets which are considered to be rich in fiber. GB diets include ground wheat, ground corn, wheat middlelings (a wheat by-product), alfalfa meal, soybean meal, and pulp of dried beet as the main ingredients. On the other hand, PIB diets are made with refined ingredients which contain key nutrients including, corn starch as carbohydrate source and casein as proteins source^8^. The American Institute of Nutrition (AIN) has developed two PIB diets namely, AIN-93 M (M for mature) and AIN-93G (G for growth and reproduction)^9^, for laboratory animals. Both GB and PIB diets used in our present study contain 5% crude fiber, but the major difference between these diet types is due to the presence of soluble fiber. The GB diet contains both soluble and insoluble fibers whereas the PIB diet contains mainly cellulose, which is an insoluble fiber^10^.

The beneficial effects of cellulose (insoluble or non-fermentable fiber) supplementation are observed in terms of altered gut microbiota composition and subsequent protection against dextran sodium sulfate (DSS)-induced colitis^11^. Additionally, by increasing the amount of long-chain fatty acids and activating mucosal and systemic Th2-immune responses, the non-fermentable fiber helps to alleviate central nervous system specific autoimmune disease^12^. However, the insoluble nature of cellulose makes it poorly fermentable by the gut microbiota in mice and rats resulting in a reduced production of SCFAs^10^. In addition, a reduced fermentation will lead to an overall low diversity of bacteria in the gut, which can have a profound impact on gut health and development of metabolic diseases^10^. Due to this, the cellulose-based purified diets with limited fermentability may lead to adverse health effects. However, the exact underlying mechanism that mediates fibers’ effects on gut health is poorly understood. But a role of the intestinal microbiota in this process cannot be ruled out.

In the present study, we have explored how the lack of soluble fiber in the PIB as compared to the GB diet affect the intestinal microbiota dynamics given the overall fiber content remains the same (5%) in the two type of diets. We used whole metagenome shotgun sequencing (WMGS) approach to unravel the gut microbiome of mice fed with GB and PIB diets for a period of two months and performed comparative metagenomic analysis to investigate the fiber type specific gut microbiota structure and function dynamics.

## Results and discussion

### Comparative taxonomic analysis of the GB and PIB diets fed mice gut metagenomes

**Figure 1:**
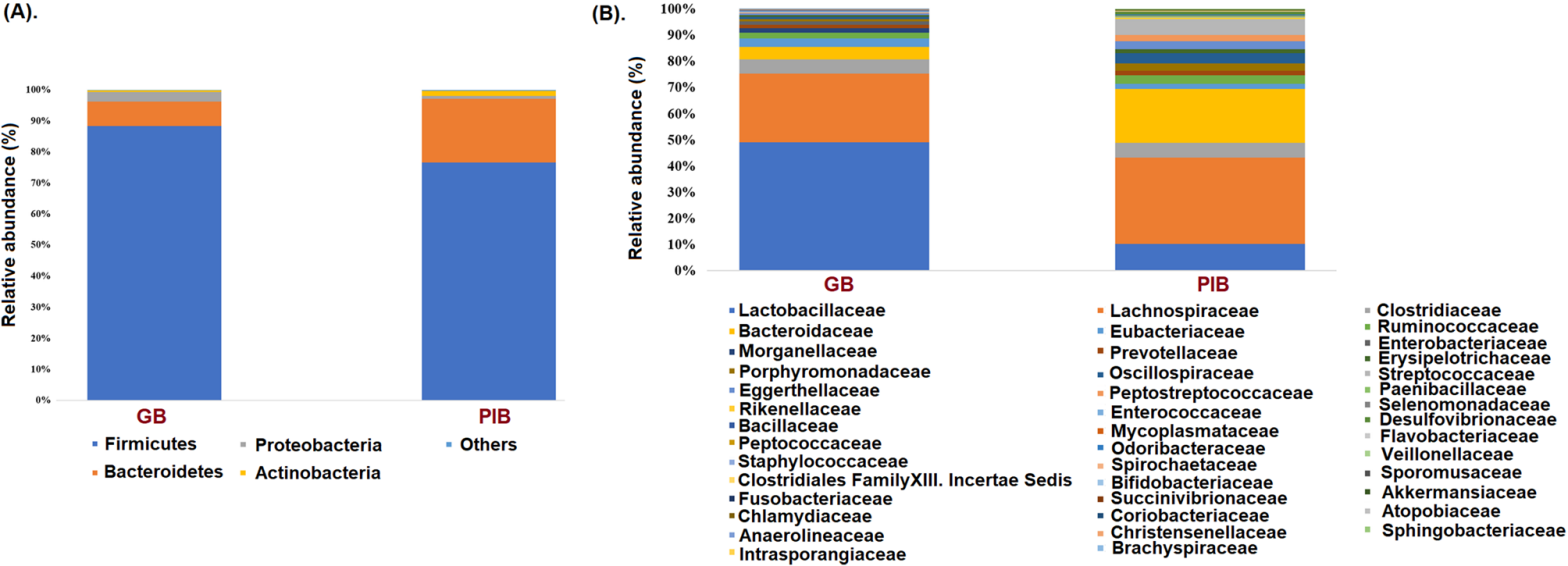
Stack bar plot represents relative abundance differences at (**A**) phylum and (**B**) family levels between GB and PIB groups.

Quality and preprocessing analysis of raw metagenomic data was done using the methods described below (Table S1). The relative abundances of microbial taxa were analyzed which demonstrated microbial community variations between the GB and PIB groups (Fig. 1). The two dominant phyla of human and mice gut namely, Firmicutes (F) and Bacteroidetes (B), clearly indicated changes in these two groups. An increased F/B ratio was found in the GB as compared to the PIB group (Fig. 1A). Some members of both these phyla are known to participate actively in the saccharolytic activities^13,14^.

**Figure 2:**
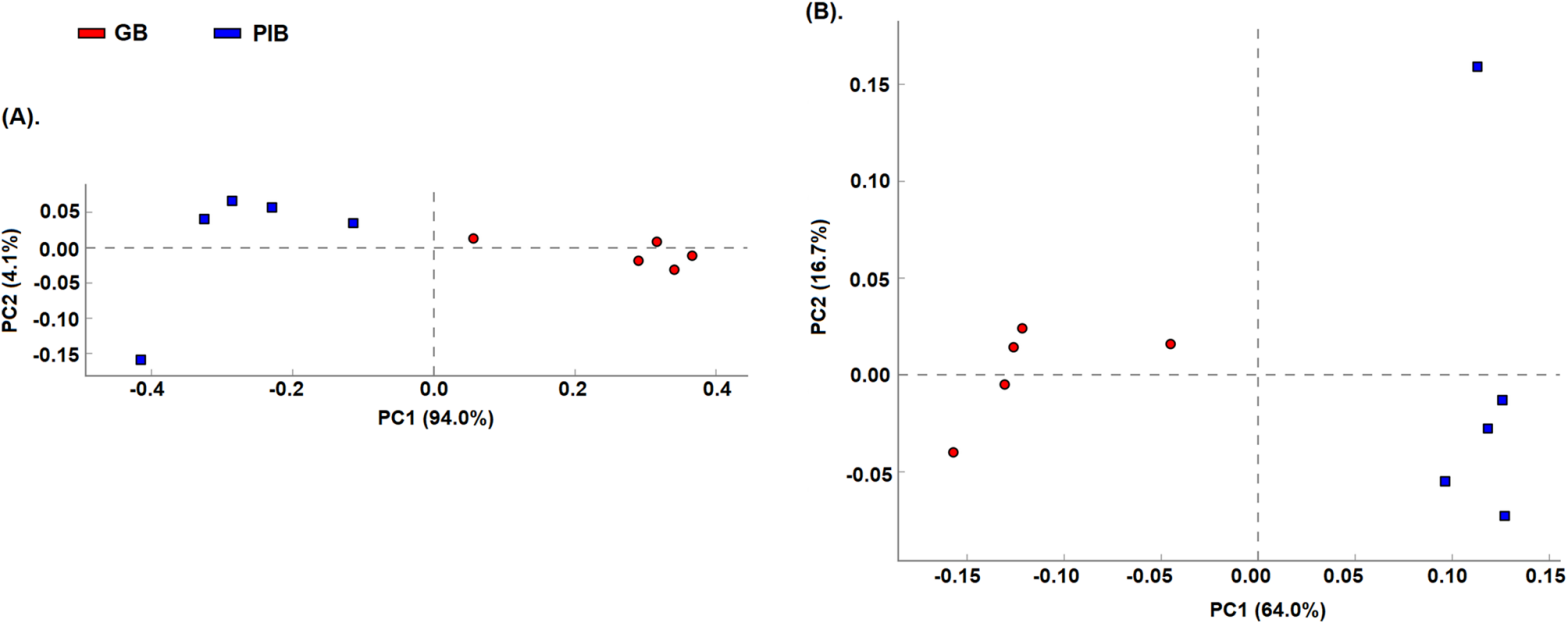
Principal component analysis (PCA) plot of the taxonomical profiles of GB and PIB groups at (**A**) genus, and (**B**) species levels. The different color reflects the fiber type, and each dot represents an individual sample. In the axes legends, the percent variability explained by PC1 and PC2 is provided in parentheses.

In the anaerobic environment of gut, the mineralization of complex organic matter occurs through a concerted action of a variety of microorganisms. Towards this, differentially abundant taxa alterations were analyzed after GB and PIB diet intake in mice at different levels of taxonomy, namely phylum, class, order, family, genus, and species. The segregation of the GB and PIB groups based on the taxonomical features was analyzed using the principal component analysis (PCA) (Figs. 2 and S1) and separate clusters comprising of the samples of the two diet groups were identified (Fig. 2A,B). Thus, the presence of soluble fiber in the GB diet is found to be associated with an altered microbiome composition as compared to the PIB diet.

Fiber degradation, fermentation, acetogenesis, sulfate reduction, and methanogenesis are the microbial processes that coexist in a variety of natural and engineered anaerobic environments^15^. The primary fermenters, such as complex polysaccharide degraders, break down the complex molecules and ferment the hydrolysis products along with the secondary fermenters. The key bacterial fermentation products are SCFAs and gases such as hydrogen (H_2_) or methane^15^. There are three main microbial routes by which the excessively produced H_2_ gas can be removed to enable the depletion of electron sink products such as lactate, succinate, and ethanol, and allow more efficient energy recovery from organic substrates^15^. These are dissimilatory sulphate reduction, methanogenesis, and acetogenesis, which can convert H_2_ into hydrogen sulfide (H_2_S), methane, and acetate, respectively. Acetogenesis has been shown to be inversely correlated with methanogenesis^16^.

**Figure 3:**
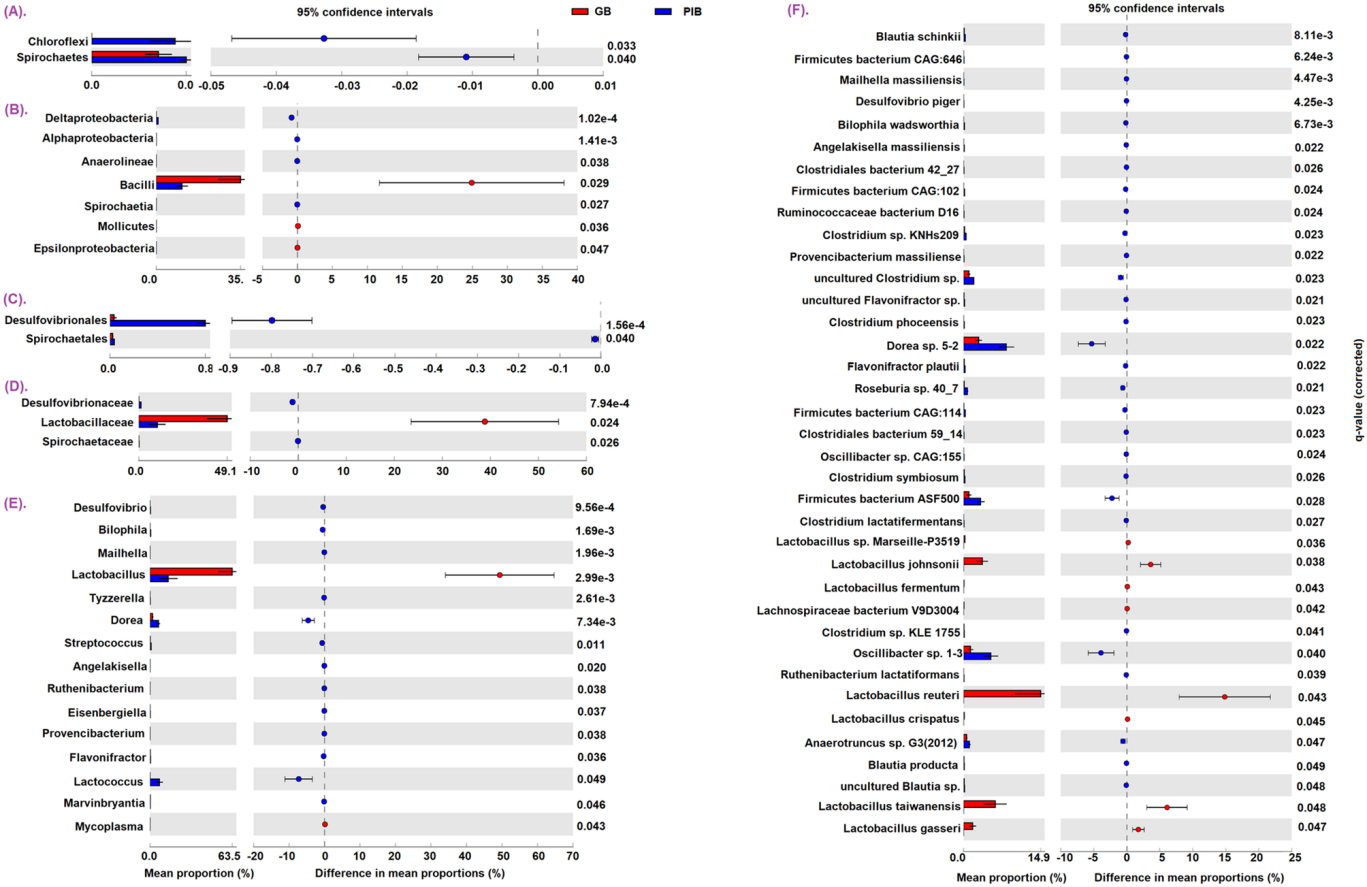
Extended error bar plot showing the differentially abundant taxa at all taxonomic levels, namely, (**A**) phylum, (**B**) class, (**C**) order (**D**) family, (**E**) genus, and (**F**) species.

In our analysis, we have identified differentially participating microbial taxa (Fig. 3) in the above-mentioned processes between the GB and PIB diet groups and the roles of these taxa in fiber degradation and other processes are summarized in Table 1. Overall, the bacterial taxa involved in cellulolytic ability are found to be statistically significantly increased in the PIB diet group as compared to the GB group*.* For example, the PIB mice group is found to have a significant enrichment of the taxa belonging to the families *Ruminococcaceae* and *Lacnospiraceae* and class *Anaerolineae*. Some members of these families and class are known to have cellulolytic ability. This is in corroboration with the fact that PIB diet harbors insoluble fiber, namely cellulose, which may be degraded by these cellulolytic bacterial taxa. Another taxa that is found to be significantly increased in the PIB group is the genus *Desulfovibrio.* It is known that the members of genus *Desulfovibrio* help in the removal of excessive H_2_ produced during the fermentation process and convert it into H_2_S. The increased volume of H_2_S gas sometimes can cause irritation in gut and can subsequently cause enteric inflammation^17^. Some studies also demonstrate a higher counts of *Ruminococcaceae* in patients of colonic Crohn's disease^18^. Taken together, these observations suggest that the PIB diet with a cellulose concentration of 5% may enhance the abundance of taxa responsible for high H_2_S production, namely *Desulfovibrio* (genus) and *Desulfovibrio piger* (species) (Fig. 3E–F) which may result in host gut inflammation.

**Table 1:** The roles of significantly increased taxa in the PIB and GB diet groups shown in Fig. 3 in fiber degradation and other processes.

Group	Phylum	Significantly altered taxa	Roles
PIB	Firmicutes *Lachnospiraceae* (family)	*Tyzzerella* (genus); *Dorea* (genus); *Eisenbergiella* (genus); *Marvinbryantia* (genus); *Clostridium lactatifermentans* (species); *Blautia producta* (species); *Blautia schinkii* (species); Clostridium sp. KNHs209 (species); *Clostridium symbiosum* (species)	*Lachnospiraceae* family is proven to have fibrolytic specialization, and possesses a large number of cellulose degradation pathways^25^. Members of *Marvinbryantia and Blautia* are acetogens^29^
PIB	Firmicutes *Ruminococcaceae* (family)	*Angelakisella* (genus)*, Angelakisella massiliensis* (species)*; Ruthenibacterium* (genus)*, Ruthenibacterium lactatiformans* (species)*; Provencibacterium* (genus)*, Provencibacterium massiliense* (species)*;* *Ruminococcaceae bacterium D16* [unclassified *Ruminococcaceae*]	*Ruminococcaceae* family is proven to have fibrolytic specialization, and possesses a large number of cellulose degradation pathways^25^
PIB	Chloroflexi	Anaerolineae (class)	Most of the isolated strains of Anaerolineae has the potential to degrade cellulose, carbohydrates, and/or proteins anaerobically, playing an important role as primary and secondary fermenters^30^
PIB	Proteobacteria	Deltaproteobacteria (class); Desulfovibrionales (order); *Desulfovibrionaceae* (family); *Desulfovibrio* (genus), *Desulfovibrio piger* (species); *Mailhella* (genus), *Mailhella massiliensis* (genus)	To remove access H_2_ gas produced during the fiber metabolization, *Desulfovibrio* probably performs dissimilatory sulphate reduction^15^. *M. massiliensis* have sulfate reducing ability^31^
GB	Firmicutes	Bacilli (class); Lactobacillales (order); *Lactobacillaceae* (family); *Lactobacillus* (genus); *L. crispatus, L. taiwannesis, L.gasseri, L. johnsoniii, L. fermentum* and *L.reuteri* (species)	Rapidly grow upon soluble fiber
GB	Firmicutes	*Lachnospiraceae bacterium V9D3004* (member of unclassified *Lachnospiraceae*)	

In the GB diet group, which contains both soluble and insoluble fiber, the members of phylum Firmicutes (mainly genus *Lactobacillus*) are predominantly observed. The species of genus *Lactobacillus* have multiple beneficial effects on host health, such as prevention and/or amelioration of diverse disorders. For example, *L. johnsonii NCC 533* is a well-known probiotic with immuno-modulatory and pathogen inhibitory functions^19^. Previous studies have also demonstrated the immuno-modulatory and anti-inflammatory properties of *L. fermentum CECT-5716*^20^. Similarly, when this probiotic was administered to colitic rats a significant reduction of IL-1β and TNF-α levels and colonic iNOS expression were observed^20^. In addition, various clinical findings in humans indicated that in both adults and children *L. reuteri* relieved abdominal pain in individuals with IBD or colitis and reduced the period of acute infectious diarrhea^21–23^. *L. reuteri* could improve dyspepsia and gastritis symptoms in patients with *Helicobacter pylori* infection^23^, enhanced intestinal motility, and alleviated severe constipation^24^. Another taxa, namely *Lachnospiraceae bacterium V9D3004* is found to be significantly increased in the GB group. The members of the *Lachnospiraceae* family have fibrolytic specialization and possess a large number of cellulose degradation pathways^25^. It is important to note that the GB diet also contains some insoluble fiber, which needs to be degraded by such fibrolytic bacteria. Taken together, these findings imply that a soluble fiber content in GB diet, even though administered at low percentage (5%), may increase the number of probiotic taxa, may have beneficial effects on host health, and thus may offer prebiotic potential.

A number of studies performed in the past have explored the effect of variable concentrations and types of dietary fiber on mice gut health and microbiome composition. For example, in a study mice were fed with diets which were fully fiber-free (FFD) and those containing 7% cellulose as the only source of dietary fiber (CD)^26^. When CD and FFD mice were compared in an experimental colitis paradigm, FFD mice showed higher vulnerability to intestinal inflammation, even at low dextran sodium sulfate (DSS, 1.5%) doses, as measured by weight loss, diarrhea, and shorter colon length. In addition, mice fed with low dietary cellulose (0.3%, LCD) diet exhibited aggravated inflammation upon DSS treatment. The high-cellulose diet fed mice (30.0% cellulose, HCD) were protective against DSS-induced colitis. This indicates towards an overall beneficial effect of dietary fiber, even though it is only insoluble fiber like 7% cellulose, as compared to the fiber-free diet. In another study, in mice fed with normal chow diet containing 6% crude fiber (soluble and insoluble), inflammation was found to be restricted to the colon’s middle and distal regions upon DSS treatment^18^. This indicates towards a partially protective effect of soluble fiber in DSS treated mice.

The gut microbiome alterations also have been observed in the above mentioned and other similar studies due to the effects of dietary fiber. In a study, Sidiropoulos et al.^27^ transplanted wild and captive douc (gut microbiomes more like Western humans than their wild counterparts) gut microbiota into germ-free mice and then exposed them to either high- (4.7% crude fiber) or low-fiber (5% cellulose) diets. Among the other taxa, decreased relative abundances of *Clostridium* and *Lactobacillus* and an increased relative abundance of *Desulfovibrio* is observed in the low-fiber fed mice. These observations are in corroboration with the altered taxa composition obtained in our analysis in the two diet groups. Interestingly, a comparison of the cecal contents of mice fed with HCD and LCD revealed higher levels of *Ruminococcaceae* and *Oscillibacter* in LCD-fed mice^18^. These taxa alterations obtained between the dietary groups could be as a result of the extremely distinct cellulose percentages (LCD: 0.3% and HCD: 30% cellulose). In addition, in the FFD mice an increase in *Porphyromonadaceae*, *Verrucomicrobiaceae*, and *Bacteroidaceae* and a decrease in the relative abundance of the families *Ruminococcaceae*, *Lachnospiraceae*, and *Desulfovibrionaceae* has been observed^26^. Interindividual differences in the gut microbiome compositions in animals obtained from different sources are commonly observed^28^.

In addition to the beneficial microbial community variations, both groups also exhibited an increased abundance of opportunistic pathobionts (Fig. 3). An overall significant enhancement of genus *Bilophila* (e.g. *Bilophila wadsworthia*) and family *Spirochaetaceae* is observed in the PIB group as compared to the GB group. A positive association of the members of the above-mentioned taxa has been previously reported in inflammatory bowel diseases (IBD)^32,33^. Similarly, the GB group is found to be significantly dominated by genus *Mycoplasma* (class Mollicutes) as compared to the PIB group. A high prevalence of *Mycoplasma pneumoniae* is observed in the intestinal mucosal biopsies from IBD patients^34^. These findings indicate that under low fiber content conditions in diet (< 5% only insoluble or soluble and insoluble) the bloom of pathogenic bacteria may be increased.

### Comparative functional analysis of the GB and PIB diets fed mice gut metagenomes

**Figure 4:**
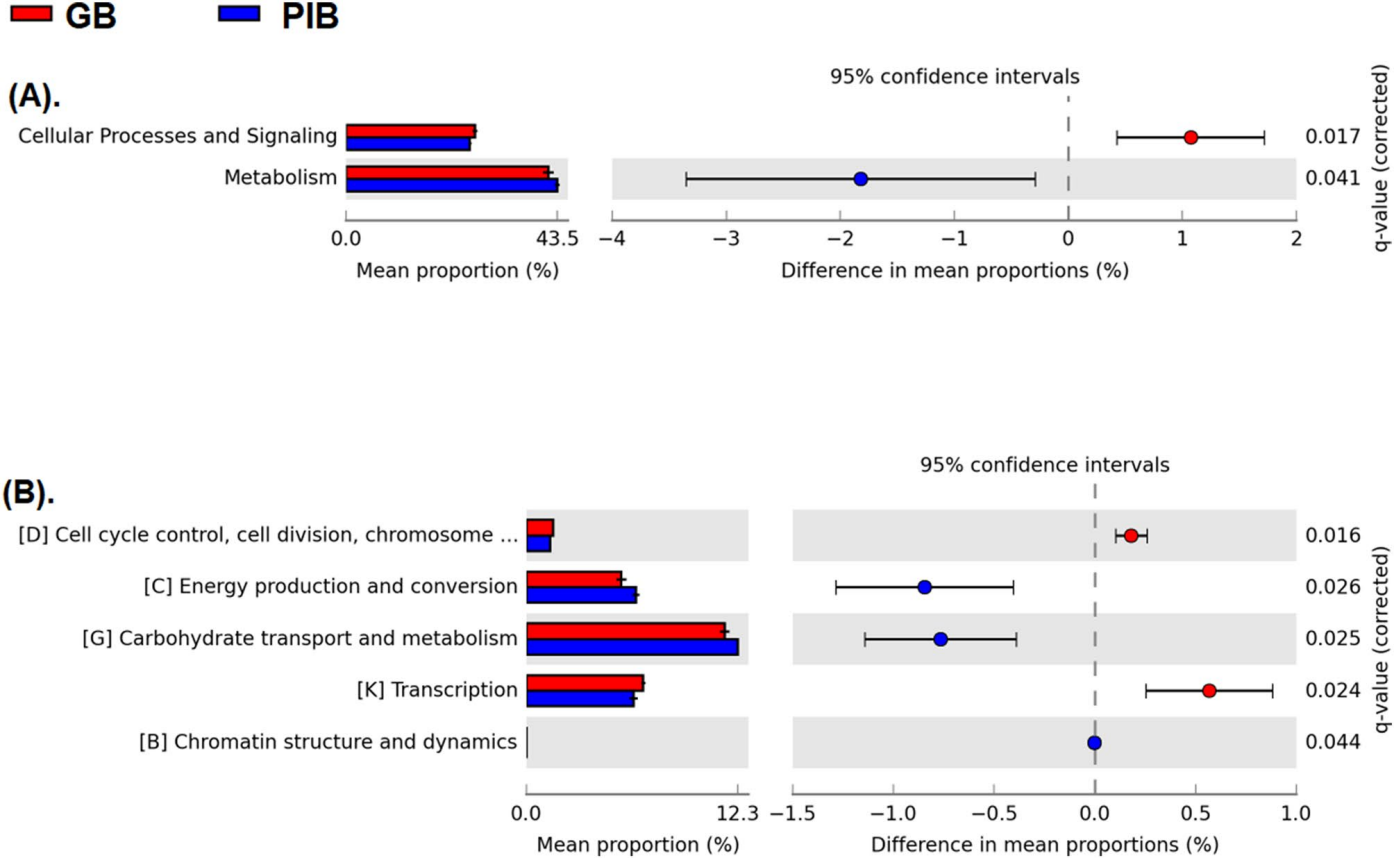
Extended error bar plot at the functional level 1 and level 2 according to the EggNOG functional hierarchy.

We identified the differentially abundant functions between the GB and PIB groups using the EggNOG based classification of the metagenomic reads into three functional hierarchies, namely, level 1, level 2, and level 3. A PCA analysis was also performed at all the three levels which revealed some separation of the samples, however, distinct clusters were not obtained (Fig. S2). At level 1, the reads mapped onto the “cellular processes and signaling” class were significantly highly abundant while those mapped onto the “metabolism” class were less abundant in the GB group as compared to the PIB group (Fig. 4A). At level 2, reads mapped onto the “cell cycle control”, “cell division”, “chromosome partitioning”, and “transcription” classes were found to be highly abundant while those mapped onto the “carbohydrate transport and metabolism”, “energy production and conversion”, and “chromatin structure and dynamics” classes were less abundant in the GB group as compared to the PIB group (Fig. 4B).

In the GB group the soluble fiber, which is also present along with the insoluble fiber, is readily available for the enteric bacterial fermentation process^35^. Thus, the microbiota may readily metabolize the soluble fiber resulting in energy production and this energy may be used in the enhancement of different energy expensive cellular processes namely, cell cycle control, cell division, chromosome partitioning and transcription. This could be one of the possible reasons for the enhancement of these functional classes in the GB group. Similarly, a higher abundance of the metabolism function in the PIB group also correlates well with the presence of a significantly higher abundance of cellulolytic microbes in this group which will metabolize the insoluble fiber (cellulose) also to produce energy.

At level 3, 980 COGs/NOGs were found to be altered between the two groups, out of which 444 were increased and 536 were decreased in the GB group as compared to the PIB group (Table S2). At level 3, we also found a similar pattern of changes as those observed at level 2. For example, at level 3 the COGs/NOGs related to “cell cycle control”, “cell division”, “chromosome partitioning”, and “transcription” were found to be more abundant while those related to “carbohydrate transport and metabolism”, and “energy production and conversion” were less abundant in the GB as compared to the PIB group.

The “soluble” classification of dietary fiber typically includes compounds including pectins, hemicelluloses, mucilages, and gums. Insoluble dietary fibers, on the other hand, include cellulose, resistant starch, and lignin^36^. Due to the difference in the nature of the fiber present in the two types of diets used in our study a difference in the enzymes responsible for the metabolization of these fiber sources is expected between the GB and PIB groups. Towards this, we explored the respective fiber degrading enzymes in the metagenomic samples from both the groups (Table 2). Interestingly, functions associated with other glycan degradation (map00511: COG0383, COG3669, COG1472, ENOG410XQYG), glycosaminoglycan (ENOG410YDJW, COG1472) degradation and glycogen debranching enzyme (COG1523) were found to be significantly increased in the PIB as compared to the GB group. As cellulose is a complex polysaccharide, whose breakdown may take sometime, the gut microbiota of the PIB group might be in nutrient stress conditions. As a result, they might target the host-derived oligosaccharides and self-stored glycogen alongwith availing the available cellulose for subsequent energy production. This might also be one of the reasons for the increased abundance of “metabolism” and “energy production” functional classes in the PIB as compared to the GB group. Furthermore, a low-fermentable-fiber diet is frequently linked to greater consumption of host-derived glycans and higher levels of *D. piger*^37^. The PIB group in our analysis also exhibited a higher abundance of *D. piger* (Fig. 3F) and host-derived glycans foraging functions.

**Table 2:** COGs/NOGs involved in the GB and PIB dietary components degradation and their subsequent transportation in bacterial cells.

Enzymes and transporters	GB	PIB
Cellulolytic enzymes	ENOG410Y1JM, COG3405	ENOG410ZVSP, COG1472, ENOG410Y3MI
Pectate lyase	ENOG410ZIM0	
Amylolytic enzyme	COG1543, COG0366, ENOG410XR0E	
Xylanase	COG3405	
PTS	COG2893, COG3715, COG1455, COG1445, COG3775, COG1080, ENOG41124SH, ENOG411253B	COG3037, COG1264, ENOG4112AIE
MFS	COG2814, COG2270, COG2211, ENOG410XP3M, ENOG410XQVS, ENOG410ZVV9, ENOG410XPZP, ENOG4111M79, ENOG410XRAD, ENOG410ZZ3F, ENOG4112APV, ENOG410XQPT, ENOG410ZVUY, ENOG410XNQK	ENOG4111JHI, ENOG41100BF, ENOG41100KC, ENOG4111RRR, ENOG411004P, ENOG410XPHE, ENOG410XQUK, ENOG410XT9M, ENOG410XRWU, ENOG410ZWMW, ENOG4111RP5
Glucose uptake	COG4975	
ABC	COG2182, ENOG410XPZR	ENOG410Y5HS, ENOG41117U2, ENOG410XS1A, COG0395, COG1653, ENOG4110K2H, ENOG4111ISI, ENOG410XPXA, ENOG41104RH, ENOG410ZWTQ, ENOG410ZVIE, ENOG410XSDP, ENOG410ZVPY
TRAP		COG4663, ENOG4111JAX, ENOG410ZWSR

The sugar transporters viz, phosphotransferase system (PTS) and major facilitators (MFS) were found to be more abundant, while ABC transporters were less abundant in the GB as compared to the PIB group (Table 2). This shows that both the groups were efficiently involved in the uptake of saccharides albeit via different mechanisms. After the uptake of saccharides, the functions contributing to the glycolysis process remained almost similar in both the groups, however, significant differences were observed in the other metabolic processes (Table 3). This indicates that when the gut microbes of the PIB group are engaged in metabolizing sugars and producing energy, those of the GB group might have already reached to the next level and enhance its replication, recombination and repair, transcription, translation, ribosomal structure and biogenesis (Table S2) and subsequently cell division (Table 4). This may be yet another reason for a higher energy production related functions in the PIB than the GB group.

**Table 3:**
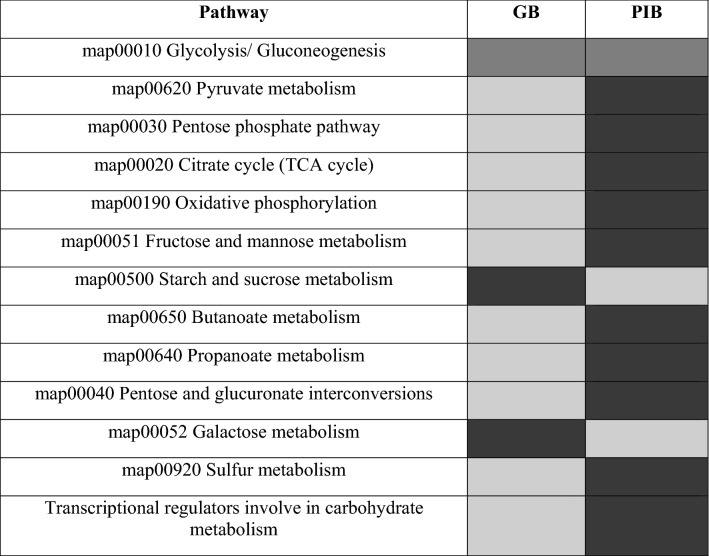
Pathways related to carbohydrate metabolism and transport and energy production and conversion in the GB and PIB groups. Light grey represents decreasing, dark grey represents increasing, and medium grey represents approximately equal functions.

**Table 4:** COGs/NOGs involved in cell cycle control, cell division, and chromosome partitioning in the GB and PIB groups.

Function	GB	PIB
Membrane attachment of FtsZ and regulation of Z-ring dynamics	FtsA (COG0849), EzrA (COG4477)	
Divisome maturation and stabilitity, peptidoglycan (PG)—binding	FtsQ (COG1589), FtsB (COG2919), DivIC (COG4839), GpsB (COG3599)	FtsQ (ENOG41124NJ)
Rod shape determining proteins	MreB (COG1077), MreD (ENOG411294E)	
PG synthesis (and its regulation)	PBP1B (COG0744), PBP2B (COG0768), murE (COG0769), murG (COG0707), ddl (COG1181), COG3757	
PG hydrolysis (and its regulation)	Amidases (COG5632, ENOG41123CS), FtsX (ENOG410YARX)	Amidases (COG3773, COG3023, ENOG41109XI, ENOG41123FY), FtsX (COG2177), FtsE (COG2884), NlpD (COG0739), NLP P60 (COG0791), Tranglycosylase (COG0741), ENOG4111U22

Interestingly, the species *L. reuteri* of genus *Lactobacillus*, which is one of the significantly abundant taxa in the GB as compared to the PIB group, is capable of attaching to the mucin and intestinal epithelia. Some strains of this species can also adhere to the gut epithelial cells in a range of vertebrate hosts^38^. A possible mechanism for this adherence is the binding of the bacterial surface molecules to the mucus layer. Mucus-binding proteins (MUBs) and MUB-like proteins, encoded by Lactobacillales-specific clusters of orthologous protein coding genes, serve as the adherence mediators or adhesins^39^. Towards this, we found a significantly increased abundance of the MucBP (MUCin-Binding Protein) domain containing COGs namely, ENOG41127KM, ENOG4111GRZ in the GB group. In addition, recently it has been illustrated that *Lactobacilli* also harbor multiple unique bile salt hydrolases as a strategy for adapting to their host niche in the intestine^40^. Gut adaptation also appears possible in the GB group due to the presence of significantly much higher abundance of bile-salt-hydrolase (COG3049) gene of *Lactobacillus spp* (Table S2).

Exopolysaccharides (EPS) produced by lactic acid bacteria serve a key role in bacterial interactions during colonization of the gastrointestinal tract. The EPS produced by *L. reuteri* are important for biofilm formation and adherence of *L. reuteri* to epithelial surfaces^41^. EPS produced by rodent *L. reuteri* 100-23 was demonstrated to induce Foxp3 + regulatory T (Treg) cells in the spleen^42^. The EPS of this strain was found to be a levan (β-2, 6-linked fructan)^42^. We found a significantly increased abundance of levansucrase EC 2.4.1.10 (ENOG410XR0E) in the GB as compared to the PIB group. Additionally, *L. johnsonii* harboured the EPS gene cluster in which epsA was proved to be essential for EPS biosynthesis^43^. We found increased functions involved in EPS biosynthesis process in the GB group, namely, the priming (also known as initiating) glycosyltransferases (ENOG410ZVIP, ENOG410ZW90, COG0438), LytTr transcriptional regulator (COG3279, ENOG410XX0T, ENOG4111WH8—product of epsA), Wzz domain (ENOG410XXQK—related with epsB), and UDP galactopyranose mutase (COG0562—product of glf). In contrast, PIB group only encompasses the priming glycosyltransferases (COG4632, ENOG4111SD1, ENOG4111JGN, ENOG4111H3C, ENOG410XRQ3, ENOG410ZWDI, ENOG410XTCA, ENOG410YB08, ENOG410XP57) and exopolysaccharide biosynthesis protein (COG4632). Therefore, the GB group comprises more functions involved in the different steps of EPS biosynthesis as compared to the PIB group.

### Taxa Co-occurrence analysis in the GB and PIB diets fed mice gut metagenomes

**Figure 5:**
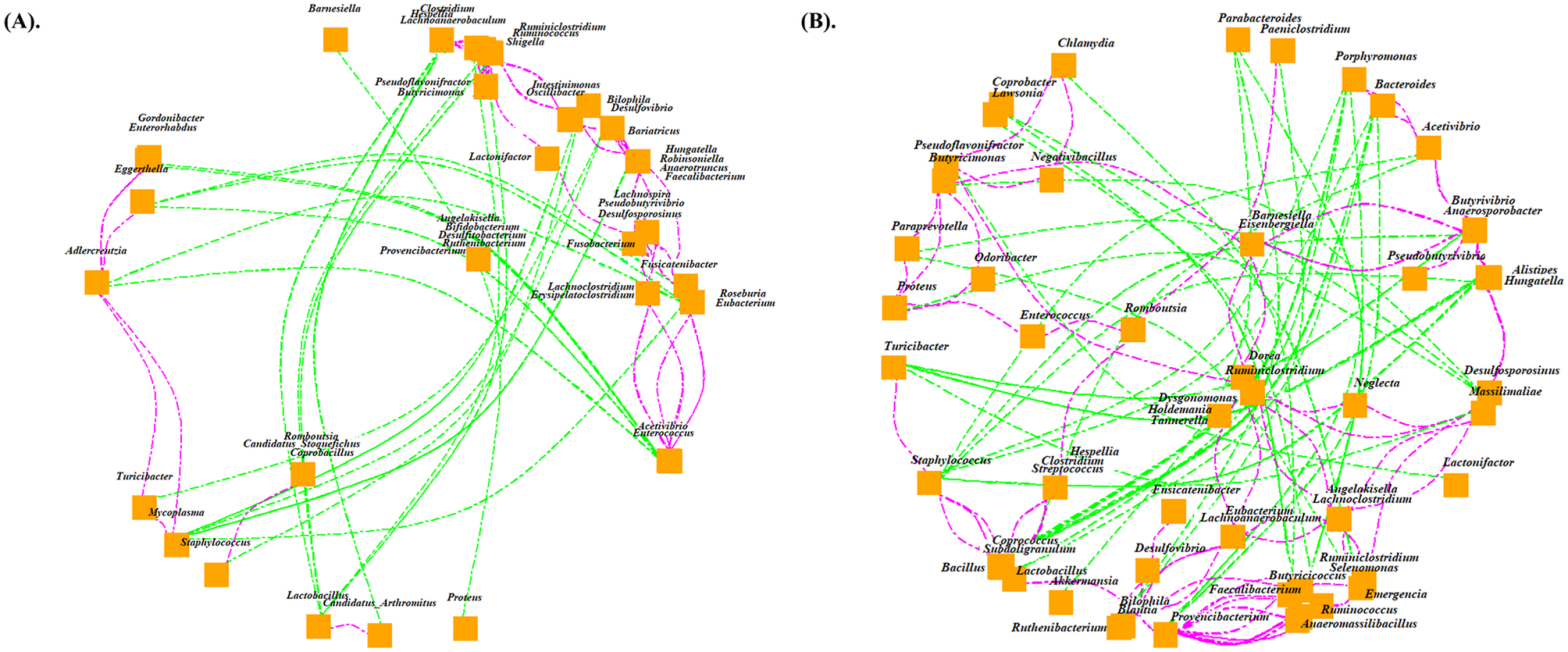
Co-occurrence analysis revealed differences in the concerted action of the gut microbial community in different diet compositions. Taxa correlation network plot in (**A**) GB and (**B**) PIB groups. Spearman correlation coefficient threshold > 0.65 and *p*-value < 0.05 is used to determine significant correlations among taxa. Magenta color represents positive and green color represents negative correlations.

As the overall fermentation process relies on a concerted effect and co-ordination of various microbial members, a co-occurrence analysis of the microbial taxa is expected to reveal the combinations of genera interacting with each other within the different groups (Fig. 5). In the GB group the significantly increased genus *Lactobacillus* is found to be in positive correlation with the segmented filamentous bacteria (SFB). In a previous study, penicillin treatment was used in germfree BALB/c mice, which did not harbour *Lactobacilli* in their GI-tracts, to explore the microbial colonization in gut^44^. These animals showed the colonization of the gram-negative anaerobes and coliforms, but not enterococci or filamentous segmented ileal microbes^44^. In another study, Ivanov et al. reported the abundance of 479 taxa to be significantly different between SFB-positive and -negative mice strains (*p* < 0.05)^45^. A higher population of *Lactobacillus* was particularly associated with SFB-positive Taconic B6 mice.

Interestingly, genus *Mycoplasma*, which is significantly increased in the GB group, showed a positive correlation with *Adlercreutzia* and *Turicibacter* in our analysis*.* A high prevalence of *Mycoplasma pneumoniae* is observed in the intestinal mucosal biopsies from IBD patients^34^. In addition, previous studies have suggested that *Turicibacter* has a strong positive association with inflammation and may play a role in the development of IBD^46^. These observations suggests that both these genera may play important roles in diseases, including IBD. In the GB group the genus *Mycoplasma* was observed in negative correlation with a majority of taxa belonging to *Lachnospiraceae*, *Ruminococcaceae, Desulfovibrionaceae,* and *Spirochaetaceae* (Table S3). Interestingly, all these taxa are found to be in positive correlation with the significantly increased taxa in the PIB group.

A majority of genera belonging to *Lachnospiraceae* and *Ruminococcaceae* were found to be positively correlated with the significantly altered taxa in the PIB group including genus *Ruminococcus*. These observations indicate that the cellulolytic bacterial community rises in the PIB group (Table S4). As expected, a positive correlation is also observed among the H_2_ consumers namely, *Desulfovibrio, Mailhella,* and *Blautia*. Furthermore, the significantly increased genera *Marvinbryantia*, *Angelakisella,* and *Flavonifractor* were found in positive correlation with *Oscillibacter* (a member of the *Ruminococcaceae* family), which is found to have a negative relationship with the colon barrier function measures^18^. The significantly increased taxa in both GB and PIB groups are identified to be in negative correlation with *Bacteroides* and *Parabacteroides* (Tables S3–S4).

It is worth noting that the functional categories [M] Cell wall/membrane/envelope biogenesis and [P] Inorganic ion transport and metabolism correlate positively with the significantly increased taxa (*Lactobacillus*) in the GB group (S3 Table), but negatively with the significantly increased taxa (*Marvinbryantia*, *Tyzzerella*, *Provencibacterium*, *Flavonifractor*) in the PIB group (Table S4). These observations highlight the functional differences between the taxa belonging to these two categories. The other interesting observation is that at level 2 we found, significantly increased “Transcription” associated functions in the GB group in addition to the significantly increased taxa namely, *Lactobacillus*, and *Mycoplasma*. The co-occurrence analysis showed that genus *Mycoplasma* is in negative correlation with “Transcription”. This result prompted us to hypothesize that the genus *Lactobacillus* may be the primary functional contributor to "Transcription."

The GB diet, which contains both soluble and insoluble fiber will encompass more microbial accessible carbohydrates (MAC) as compared to the PIB diet which contains only insoluble fiber. Therefore, the gut microbiota of the group of mice fed with GB diet is expected to metabolize the available soluble fibers rapidly and eventually lead to flourishing of the probiotic community of microbes. Towards this, our taxonomic analysis reveals a significantly higher enrichment of probiotic *Lactobacilli* in the GB group as compared to the PIB group. In contrast, the microbiota of the PIB group is expected to metabolize the available insoluble fiber and as a result a significant increase in the cellulolytic microbes is observed in this group.

### Significant functions exhibited by significant bacteria in the GB and PIB diet groups

The taxonomic and functional analyses highlighted the significantly altered taxa and functions in the two diet groups. Next, we explored as to which significant functions were probably exhibited by which significant taxa by performing a backtracing analysis of significantly altered species and functions. Another advantage of the back-tracing analysis is to obtain the significantly important strain level taxa information. In the PIB group, out of 536 functions, 397 were backtraced and most of these mapped on *Bilophila wadsworthia 3_1_6*, *Desulfovibrio piger ATCC 29098*, *Clostridium symbiosum WAL-14163*, and *Ruminococcaceae bacterium D16* (Table S5). In the GB group, out of 444 functions, 371 were backtraced and most of these mapped on *L. crispatus ST1*, *L. fermentum CECT 5716*, *L. gasseri ATCC 33323*, *L. johnsonii NCC 533* and *L. reuteri 100–23* (Table S6). These findings imply that *Lactobacilli*, a probiotic community of microorganisms, are the main functional contributors in the gut of GB diet-fed mice, whereas gut microbiome of the PIB group of mice harbored pathobionts along with commensals.

In previous studies, the species of *Lactobacilli* were observed to be capable of attaching to mucin and intestinal epithelial cells using MucBP^38^. Towards this, the COGs ENOG4111GRZ was found to be backtraced on *L. reuteri 100–23*, *L. fermentum CECT 5716*, *L. gasseri ATCC 33323*, and *L. johnsonii NCC 533*. These observations indicate that along with *L. reuteri,* all the above-mentioned species of *Lactobacilli* play important roles in mucus binding and keeping on segregating the pathobionts from invading the intestinal barrier. Additionally, bile salt hydrolase gene associated COG3049 was found to be backtraced on *L. reuteri 100-23*, *L. fermentum CECT 5716*, *L. gasseri ATCC 33323*, *L. johnsonii NCC 533*, and *L. crispatus ST1.* We found an overall enhanced community of the *Lactobacillus* genus and the associated species and enhanced cellular processes and signaling related functions in the GB diet fed mice group. *Lactobacillus* spp. are one of the most widely used probiotics and can be found in a large variety of food products throughout the world^47^. The genus *Lactobacillus* comprises a large heterogeneous group of Gram-positive, nonsporulating bacteria which include *L. crispatus*, *L. gassari*, *L. johnsonii*, *L. taiwannesis*, *L. fermentum* and *L. reuteri*. This genus plays a very important role in food fermentation and can also be found in the GI system of humans and animals in variable amounts depending on the species, age of the host, or location within the gut^48^. These findings imply that a soluble fiber content in GB diet may increase the number of probiotic taxa which may inturn have beneficial effects on host health.

In contrast, the genera related to *Lachnospiraceae* and *Ruminococcaceae* were found to be increased in the PIB diet fed mice group. Both of these families have fibrolytic specialization and possesses complete cellulose degradation pathways^25^. In gut, methanogens, acetogens and sulphate-reducing bacteria (SRB) are able to consume the H_2_ gas produced during the fermentation process^16^. SRB can use H_2_ gas as an electron acceptor to produce H_2_S gas, thus competing with the hydrogenotrophic methanogens, but they can also grow syntrophically with some methanogens on lactate^49^. These observations corroborated with an increased abundance of acetogenes and SRB in the PIB group. However, a diet with low levels of fermentable carbohydrates is often associated with increased utilization of host-derived glycans and increased levels of *D. piger*^37^. In our analysis, we also observed an increased abundance of *D. piger* and host-derived glycans foraging functions in the PIB group. Taken together, these observations suggest that the presence of only insoluble fiber at low concentration of 5% in diet may enhance the abundance of taxa responsible for host-derived glycans foraging and high H_2_S production.

Diet has a significant impact on the composition and functions of the gut microbiota, which play critical roles in host physiology and health, including the preservation of the colonic mucus layer, which serves as a physical barrier between host and trillions of gut residents. Previous studies have shown health promotional effects of both soluble and insoluble fibers upon inducing microbiota alterations, however the fiber percentages have been higher for any visible beneficial effects^10,11,18,50^. In the present study, we elucidated the effect of GB diet containing both soluble and insoluble fibers and PIB diet containing only insoluble fiber, namely cellulose. Although the fiber content in both diet types is the same (5%), the presence of soluble fiber only in the GB diet differentiates it from the PIB diet. Our study clearly demonstrates that even at such a low percentage of dietary fiber, the presence of soluble fiber in the GB diet is associated with a completely altered microbiota composition and function as compared to the PIB diet, which lacks soluble fiber.

## Conclusion

Food is a basic requirement for survival and well-being. Diet, on the other hand, is necessary for development, health, and reproduction and plays a major role in modulating the gut microbiota. Gut bacteria are shaped by the type, quality, and origin of food, which influences their composition and function, as well as host-microbe interactions. The dietary fibers interact directly with the gut microbes and lead to the production of key metabolites such as SCFAs. In this study, we explored how the presence of soluble and insoluble dietary fiber impacts gut microbial ecology and dynamics using whole metagenome shotgun sequencing approach. Our overall findings suggest probiotic community of microorganisms, are the predominant functional contributors in the gut of mice fed with the GB diet, which contains a mixture of soluble and insoluble fiber. However, the gut microbiome of the mice fed with the PIB diet, which contains only insoluble fiber cellulose, harbors pathobionts, together with commensals, as the significant contributors. As a result, at almost the identical fiber proportion, although in low amounts (5% only), the presence of soluble fiber in the diet might affect the gut microbial ecology more favorably than the insoluble fiber alone. However, some contributions of the types of the soluble and insoluble fibers and the other ingredients present in the two diet types in the effects observed in our study cannot be ruled out. Further experimental investigations are required to confirm the outcomes of the presented work. The major limitation of our work lies in including a small number of samples in our analysis. In addition, in future experiments the measurement of gut health after the administration of different diets can better highlight the differential effects of the two diet types included in our study.

## Materials and methods

### Animal experiments and sample collection

All animal experiments were approved by the Institutional Animal Care and Use Committee (IACUC) of the RIKEN Yokohama Branch. Mice were maintained under specific pathogen-free (SPF) conditions in the animal facility at the Yokohama City University. We purchased 10 mice from CLEA Japan, inc. and these mice were divided randomly into two cages of 5 mice each. 12-week-old male SPF (C57BL/6) mice were fed with AIN-93G (purified ingredients-based) or CA-1 (grain-based) diet purchased from CLEA Japan, inc. for two months. For fecal sample collection, we put each mice in an autoclaved sterilized empty cage and waited for 10 min, during which time we collected fresh fecal samples. The autoclaved sterilized empty cage was changed for each mouse. A total of ten fresh fecal samples were collected from five AIN-93G and five CA-1 fed mice. The fecal samples were stored at −80 °C before DNA extraction.

### DNA extraction

Fecal DNA extraction was performed as described previously^51^. Briefly, 10 mg of freeze-dried fecal samples were disrupted with 3 and 0.1 mm zirconia/silica beads by vigorous shaking (1500 r.p.m. for 5 min) using a Shake Master (Biomedical Science) suspended in DNA extraction buffer containing 200 μL of 10% (w/v) SDS/TE (10 mM Tris–HCl, 1 mM EDTA, pH8.0) solution, 400 μL of phenol/chloroform/isoamyl alcohol (25:24:1), and 200 μL of 3 M sodium acetate. After centrifugation, bacterial genomic DNA was purified by the standard phenol/chloroform/isoamyl alcohol protocol. RNAs were removed from the sample by RNase A treatment.

### Whole metagenomic shotgun sequencing (WMGS) and read quality improvement

The complete workflow of the metagenomic analysis is provided as Fig. S3 WMGS sequence libraries were developed using the Illumina TruSeq DNA Sample Preparation kit with catalog number PE-940-2001. Sequencing was carried out using the Illumina HiSeq2000 platform to produce paired end reads of 126 bp. In a step of end repair, the fragments were purified using AMPureXP beads with gel-free method. Using FastQC (https://www.bioinformatics.babraham.ac.uk/projects/fastqc/), the accuracy of raw reads was analyzed. The removal of the adapter sequences was performed using FaQCs (v1.34)^52^ and the reads with an average Q-score below 30 were also removed using this software. Finally, the reads mapped on host DNA were eliminated using Bowtie2 (v2.2.5)^53^.

### Metagenomic data analysis

The metagenomic data processing and analysis follow same pipeline as previous^54^. Briefly, metagenomic content analysis was carried out using the MEGAN Community Edition (v6.8.18)^55^. For this, firstly, the filtered reads were aligned against nr-db (as of 2017) at default parameters using BLASTX option of DIAMOND (v0.9.9.110)^56^. The resultant BLASTX files were then introduced into MEGAN6 and taxonomic and functional binning of the reads was performed. LCA algorithm was used to analyze the data and to generate data summaries based on different NCBI taxonomic levels, viz, phylum, class, order, family, genus, and species. For this, the parameters chosen were minimum bit score (50) and minimum support (50). Ultimately reads get assigned to a taxonomic and functional category. The samples were normalized with respect to the smallest dataset. Only taxa or functions with a mean relative abundance > 10 counts were considered for further analysis.

The statistically significant differences between the grain-based (GB) and purified ingredients-based (PIB) diet fed metagenomic samples were identified using STAMP (v6.8.18)^57^. The differences between these two groups*,* or datasets, were analyzed using Welch’s t-test. Multiple corrections were done using Banjamini-Hochberg method. The confidence interval and *p*-value threshold for the analysis was set to 95% and < 0.05, respectively. The statistically significant functions were backtraced on statistically significant taxa using EggNOG 4.5.1^58^. Co-occurrence analysis was carried out using the Spearman correlation method within groups using R libraries Hmisc (v4.5.0) and Matrix (v1.2.18). The positive or negative association between taxa was drawn using R igraph (v1.2.6) library. A correlation coefficient threshold > 0.65 and *p*-value < 0.05 was used to determine significant correlations among taxa.

### Animal study approval

All mice experiment procedures were approved by the Institutional Animal Care and Use Committee (IACUC) of the RIKEN Yokohama Branch and abide to all regulatory standards of IACUC of the RIKEN Yokohama Branch. We hereby confirming the study was carried out in compliance with the ARRIVE guidelines.

## Supplementary Information

Supplementary Figure S1.

Supplementary Table S1.

Supplementary Figure S2.

Supplementary Table S2.

Supplementary Figure S3.

Supplementary Table S3.

Supplementary Table S4.

Supplementary Table S5.

Supplementary Table S6.

Supplementary Figure Legends.

## Data Availability

Metagenomic samples are available on NCBI having Bioproject ID PRJNA655594.
