# Supplementary figures and images for: Shotgun metagenomic sequencing revealed the prebiotic potential of a grain-based diet in mice
Source: Sci Rep. 2022 Apr 25;12:6748. doi: 10.1038/s41598-022-10762-3 (PMC9038746; doi:10.1038/s41598-022-10762-3)

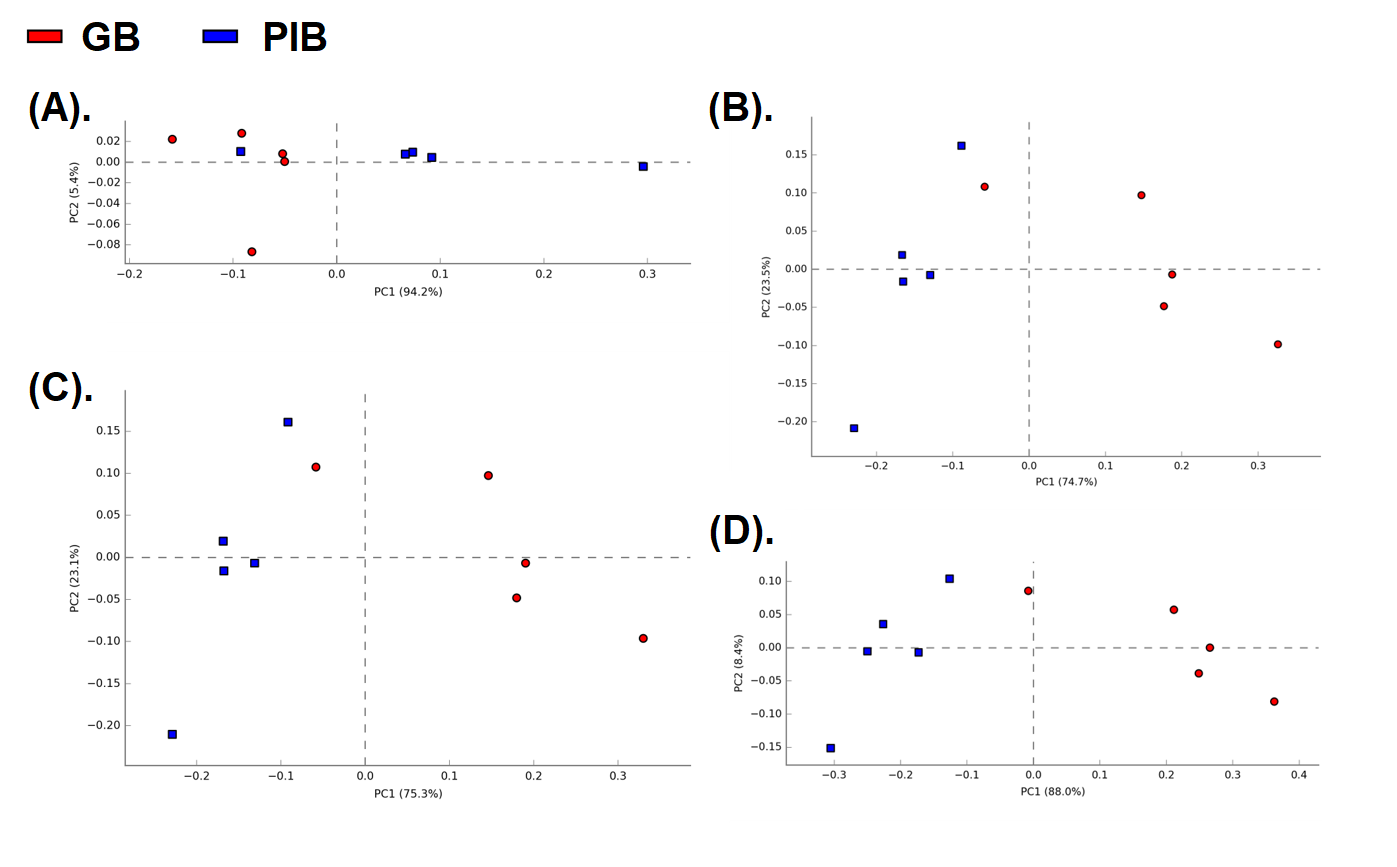

Supplement: Supplementary file 1 — Supplementary Figure S1. [file 41598_2022_10762_MOESM1_ESM.tif]

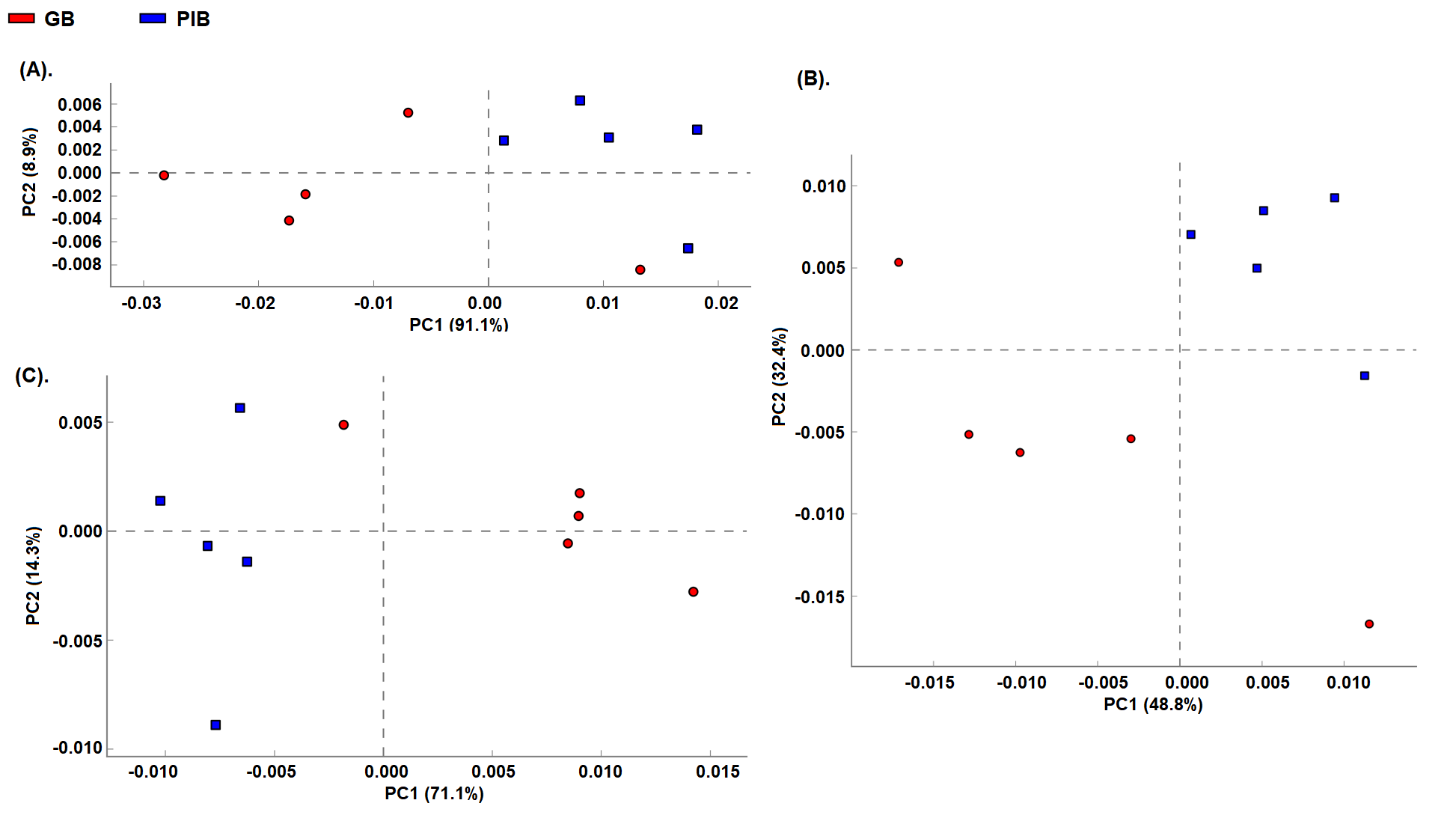

Supplement: Supplementary file 3 — Supplementary Figure S2. [file 41598_2022_10762_MOESM3_ESM.tif]

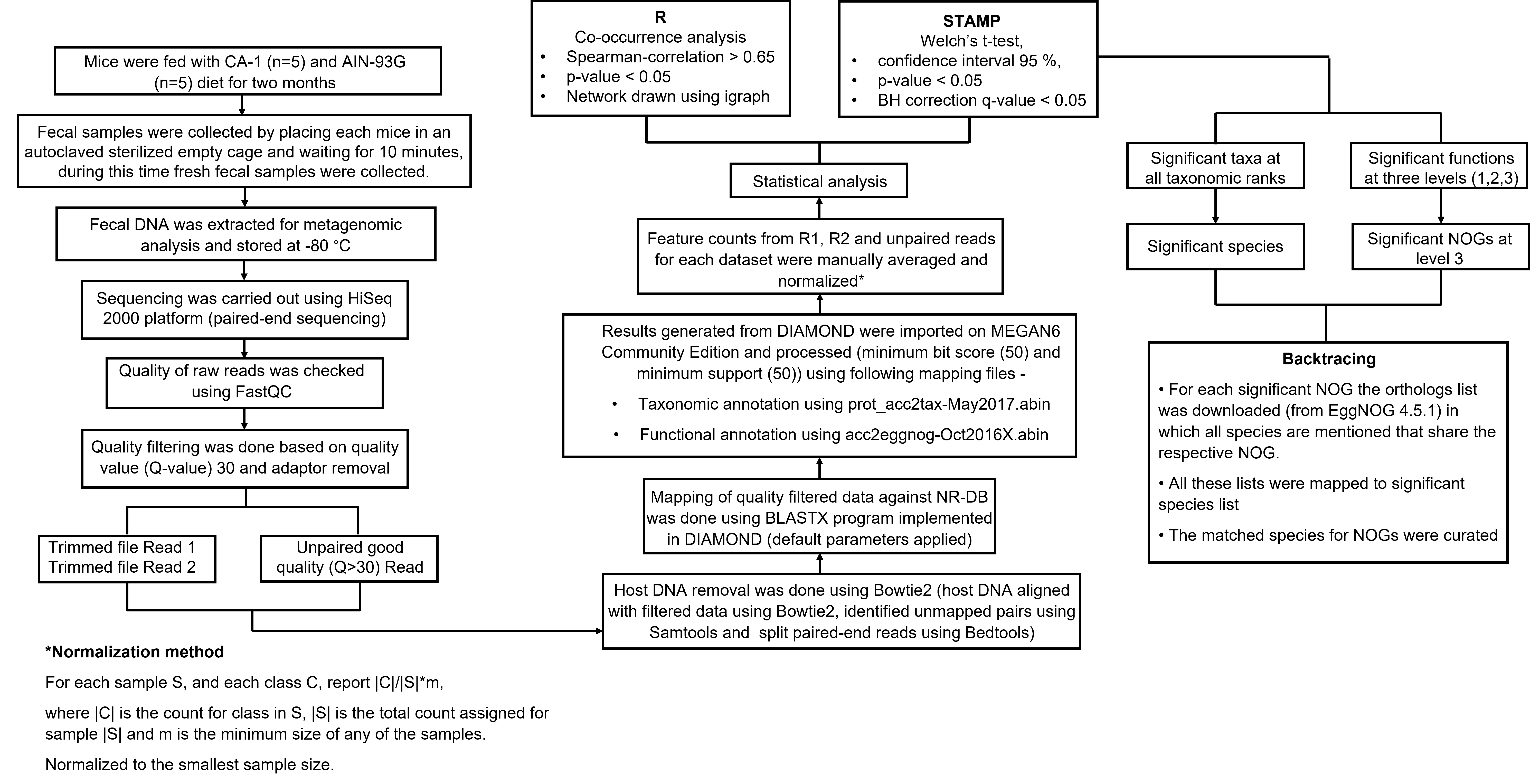

Supplement: Supplementary file 5 — Supplementary Figure S3. [file 41598_2022_10762_MOESM5_ESM.tif]
