# Supplementary material for: Shotgun metagenomic sequencing revealed the prebiotic potential of a grain-based diet in mice
Source: Sci Rep. 2022 Apr 25;12:6748. doi: 10.1038/s41598-022-10762-3 (PMC9038746; doi:10.1038/s41598-022-10762-3)
Supplement: Supplementary file 10 — Supplementary Figure Legends. [file 41598_2022_10762_MOESM10_ESM.docx]

**Titles of supplementary figures**

**S1 Fig.** Principal component analysis (PCA) plots of the taxonomical profiles of the GB and PIB groups at the (A) class, (B) order, (C) genus, and (D) species levels.

**S2 Fig.** Principal component analysis (PCA) plots of the microbiota functional profiles of the GB and PIB groups at the (A) level-1, (B) level-2, and (C) level-3 EggNOG hierarchies.

**S3 Fig.** Detailed workflow for metagenomic data processing and analysis.
